# Decoding *Blastocystis*‐Driven Mechanisms in Gut Microbiota and Host Metabolism

**DOI:** 10.1002/advs.202416325

**Published:** 2025-04-04

**Authors:** Lei Deng, Kevin SW Tan

**Affiliations:** ^1^ Shanghai Veterinary Research Institute Chinese Academy of Agricultural Sciences Shanghai 200241 China; ^2^ Broad Institute of MIT and Harvard Cambridge MA 02142 USA; ^3^ Center for Computational and Integrative Biology Massachusetts General Hospital Boston MA 02134 USA; ^4^ Laboratory of Molecular and Cellular Parasitology Healthy Longevity Translational Research Programme and Department of Microbiology and Immunology Yong Loo Lin School of Medicine National University of Singapore Singapore 117545 Singapore

**Keywords:** blastocystis, cardiometabolic health, gut microbiota, immune modulation, tryptophan metabolism

## Abstract

*Blastocystis*, a prevalent eukaryotic microorganism in the gut microbiota, has emerged as a potential link between healthy diets and improved cardiometabolic health. Despite its genetic diversity and varied host interactions, *Blastocystis* is consistently associated with healthier dietary patterns and reduced risk of cardiometabolic diseases. Current evidence suggests that *Blastocystis* may influence host metabolism by modulating gut microbial composition, short‐chain fatty acids (SCFAs) production, and immune cell differentiation. Moreover, its role in tryptophan metabolism provides intriguing insights into its potential impact on host signaling pathways. However, mechanistic evidence connecting *Blastocystis* to improved metabolic health remains limited. This perspective explores plausible pathways, including SCFAs‐mediated signaling, tryptophan metabolism, and immune modulation, through which *Blastocystis* may exert its effects. A systematic research framework integrating axenic cultivation, in vitro co‐culture systems, animal models, and multi‐omics approaches is proposed to further elucidate these mechanisms and expand the understanding of *Blastocystis* in gut health and disease.

## Graphical abstract

*Blastocystis*, a prevalent gut microbe, interacts with the host through metabolic and immune pathways, influencing cardiometabolic health. This perspective explores its role in modulating short‐chain fatty acid production, tryptophan metabolism, and immune signaling, potentially impacting cholesterol absorption, glucose metabolism, and vascular health. By integrating microbiome interactions, this work provides insights into its role as an ecosystem modulator and potential therapeutic target.

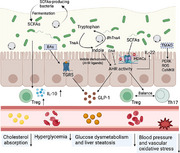

## 1 Introduction

*Blastocystis* is a common eukaryotic microorganism residing in the human gut microbiota, characterized by its extraordinary genetic diversity and wide host range.^[^ ^1^ ^]^ To date, over 44 distinct subtypes (STs) have been identified, each exhibiting substantial genetic variability and unique interactions with host organisms.^[^ ^2^ ^]^ Despite its prevalence, *Blastocystis* has been historically understudied, often regarded as a potential pathogen due to its association with gastrointestinal symptoms in some individuals.^[^ ^3^ ^]^ However, emerging evidence has challenged this notion, positioning *Blastocystis* as a potentially beneficial member of the gut microbiome, especially in the context of dietary and cardiometabolic health.

Recent large‐scale microbiome studies have provided new insights into the ecological and functional significance of *Blastocystis*. A global analysis of gut microbiota samples from 56989 individuals across 32 countries has revealed that *Blastocystis* prevalence and subtype distribution are strongly influenced by geography, diet, age, and lifestyle factors.^[^ ^4^ ^]^ Its highest prevalence has been observed in Oceania (56.29%), with the lowest in North America (6.64%), accompanied by distinct ethno‐geographical patterns in subtype distribution. Dietary habits also play a critical role, as multiple independent datasets, including the PREDICT cohorts and datasets from Italy and the U.S., have demonstrated a higher prevalence of *Blastocystis* among vegetarians and individuals adhering to a plant‐based diet. Despite country‐specific variations, a consistent pattern has emerged linking *Blastocystis* colonization to favorable cardiometabolic biomarkers, including higher high‐density lipoprotein levels, lower triglycerides, and lower systemic inflammation biomarkers (GlycA). These findings suggest that while *Blastocystis* prevalence is shaped by geographic and dietary influences, its association with improved metabolic health appears to be a robust and generalizable phenomenon across diverse populations.

Furthermore, an intervention study involving 1124 healthy participants in a six‐month personalized dietary program demonstrated significant improvements in the healthy plant‐based dietary index and the healthy eating index.^[^ ^4^ ^]^ Following the dietary intervention, both *Blastocystis* prevalence and relative abundance increased, with higher fiber and fat intake—rather than protein or carbohydrate consumption—showing strong associations with *Blastocystis* carriage. Remarkably, *Blastocystis*‐positive individuals experienced more pronounced body mass index (BMI) reductions, suggesting its potential role in promoting weight loss.^[^ ^4^ ^]^ Additionally, *Blastocystis* has been linked to favorable postprandial glucose metabolism, reduced visceral fat, and improved cardiometabolic outcomes.^[^ ^5^ ^]^ These associations suggest that *Blastocystis* may influence microbial ecology and host metabolic pathways, yet the underlying mechanisms remain poorly understood. This knowledge gap calls for targeted investigations to elucidate how *Blastocystis* interacts with its host and contributes to metabolic health.

This perspective aims to bridge these gaps by examining potential mechanisms through which *Blastocystis* influences host metabolism. These include modulation of short‐chain fatty acids (SCFAs) production, regulation of tryptophan metabolism, and immune system interactions. By leveraging advanced tools such as axenic cultivation, in vitro co‐culture systems, animal models, and multi‐omics approaches, we propose a structured research framework to systematically explore these mechanisms. Understanding the role of *Blastocystis* in host‐microbiota interactions will not only deepen our knowledge of its biological functions but also pave the way for novel dietary or microbial interventions to improve cardiometabolic health.

## 2 Key Mechanisms Linking *Blastocystis* to Host Metabolism

### 2.1 SCFAs as Mediators of Metabolic Health

**Figure 1:**
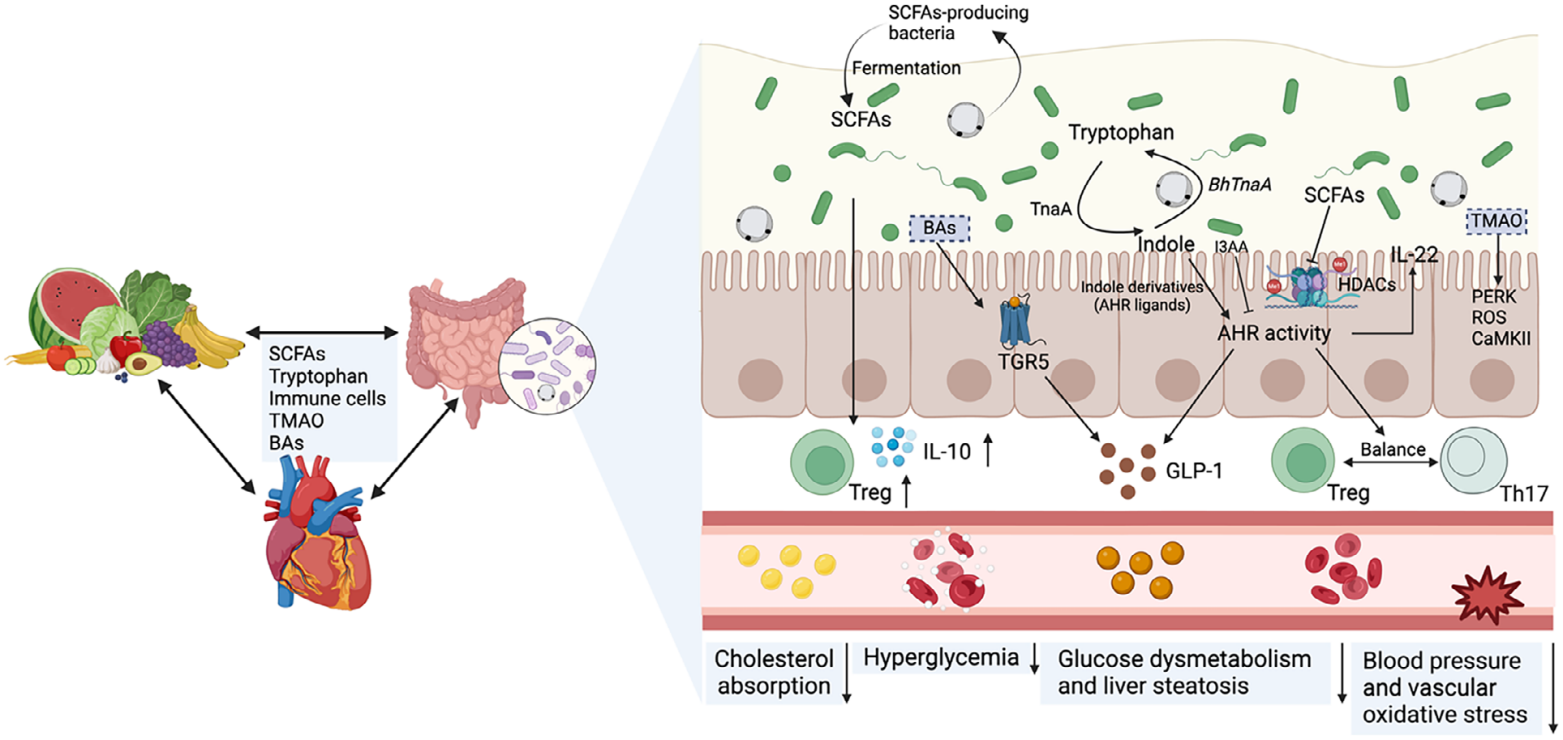
Potential mechanisms of *Blastocystis*' role in cardiometabolic health. Specific dietary components, such as fibers and proteins, affect the production of various metabolites, including short‐chain fatty acids (SCFAs), tryptophan metabolites, bile acids (BAs), and trimethylamine N‐oxide (TMAO), by the gut microbiota. These metabolites influence the local intestinal physiology and engage in crosstalk with the host, potentially altering homeostasis in various disease states. *Blastocystis* may impact host metabolism by altering SCFA production, which has been associated with cholesterol absorption, inflammation reduction, and glucose homeostasis. Additionally, *Blastocystis* encodes a unique tryptophanase (*BhTnaA*), which converts indole into tryptophan, potentially influencing aryl hydrocarbon receptor (AhR) signaling and metabolic pathways. *Blastocystis* also modulates immune responses by regulating the balance between regulatory T (Treg) and T helper 17 (Th17) cells, which are implicated in vascular inflammation and metabolic disorders. Created with Biorender.com.

SCFAs, including acetate, propionate, and butyrate, are key microbial metabolites produced by the gut microbiota through the fermentation of dietary fibers. These SCFAs are essential not only for maintaining gut health but also for exerting beneficial effects on various cardiometabolic diseases (**Figure** **1**).^[^ ^6^ ^]^ For example, the administration of propionic acid has been shown to reduce total blood cholesterol and low‐density lipoprotein levels in both human studies and animal models. This reduction is achieved through an increase in regulatory T (Treg) cells and interleukin‐10 (IL‐10) levels within the intestinal microenvironment, which subsequently suppresses the expression of Niemann‐Pick C1‐like 1, a key cholesterol transporter in the intestine.^[^ ^7^ ^]^ Additionally, supplementing germ‐free *Apoe^−/−^* mice with the butyrate‐producing bacterium *Roseburia intestinalis*, in conjunction with dietary plant polysaccharides, resulted in a fourfold increase in cecal butyrate production compared to the non‐supplemented group. This was accompanied by a significant reduction in systemic inflammatory markers, including tumor necrosis factor α (Tnf‐α) and vascular cell adhesion molecule 1, as well as a marked attenuation of atherosclerosis.^[^ ^8^ ^]^ Similarly, administration of *Intestinimonas butyriciproducens*, another butyrate‐producing bacterium, has been shown to significantly reduce body weight gain, improve hyperglycemia, decrease fat accumulation, and alleviate white adipose tissue inflammation in mice fed a high‐fat diet. These metabolic benefits are strongly correlated with a nearly twofold increase in butyrate levels in both the gut and circulation compared to the placebo group.^[^ ^9^ ^]^

While direct evidence linking *Blastocystis* to SCFAs‐mediated cardiometabolic health is limited, emerging findings suggest a potential role for *Blastocystis* in modulating SCFAs production. Notably, our prior research has demonstrated that colonization with *Blastocystis* ST4 is associated with a two–three‐fold increase in SCFA concentrations compared to non‐*Blastocystis* colonized control mice, and elevated levels of SCFAs‐producing bacteria in experimentally induced colitis mouse models.^[^ ^10^ ^]^ Furthermore, fecal microbiota transplantation (FMT) from *Blastocystis* ST4‐colonized mice into both wild‐type and Rag1^−/−^ mice resulted in significantly elevated SCFA levels, promoting faster recovery in dextran sulfate sodium (DSS)‐induced intestinal inflammation. These findings indicate that the rise in SCFA concentrations following ST4 colonization may be sufficient to confer health benefits,^[^ ^10a^ ^]^ suggesting a plausible mechanism through which *Blastocystis* may influence host cardiometabolic health. However, the long‐term persistence of these effects and their broader implications for cardiometabolic health remain uncertain. Future investigations should aim to establish causality, elucidate the molecular mechanisms underlying *Blastocystis*‐driven SCFA modulation, and explore potential therapeutic applications for metabolic disease prevention and treatment.

### 2.2 Tryptophan Metabolism and Host Signaling

Dietary tryptophan serves as a precursor for specific microbiota‐derived metabolites such as indole, indole acetic acid, and tryptamine, which are known to activate signaling through the aryl hydrocarbon receptor (AhR).^[^ ^11^ ^]^ Notably, individuals exhibiting metabolic risk factors—such as obesity, Type 2 diabetes, and high blood pressure—tend to display lower concentrations of these AhR ligands.^[^ ^12^ ^]^ Studies have shown that supplementing *Lactobacillus reuteri*, a bacterium with a high capacity for producing AhR ligands, along with an AhR agonist, can alleviate both diet‐ and genetically induced metabolic impairments, particularly glucose dysmetabolism and liver steatosis. These benefits are achieved through enhanced intestinal barrier function and increased secretion of the incretin hormone glucagon‐like peptide 1 (GLP‐1) from intestinal enteroendocrine cells.^[^ ^13^ ^]^ Furthermore, a large‐scale, longitudinal study by EPIC‐Norfolk has demonstrated that higher levels of tryptophan, as opposed to kynurenine and serotonin, are inversely associated with mortality and cardiovascular disease risk.^[^ ^14^ ^]^

Interestingly, our previous research identified a unique tryptophan metabolism pathway in *Blastocystis*, enabled by a horizontally acquired tryptophanase gene (*BhTnaA*) (Figure 1).^[^ ^15^ ^]^ TnaA is a prokaryotic enzyme typically responsible for converting tryptophan into indole, a compound that plays a pivotal role in modulating host physiology, immunity, and gut homeostasis.^[^ ^16^ ^]^ Unlike conventional bacterial tryptophanases, which degrade tryptophan into indole, *BhTnaA* catalyzes the reverse reaction, converting indole back into tryptophan. Structural comparisons revealed a significant divergence between *BhTnaA* and its homolog in *Escherichia coli* K12, yet this enzyme remains highly conserved across *Blastocystis* ST1, ST4, and ST7. While functional studies on *BhTnaA* knockout mutants are currently lacking, enzymatic assays confirm that *BhTnaA* is the sole enzyme in *Blastocystis* responsible for this unique tryptophan metabolism. Kinetic analyses further indicate that *BhTnaA* exhibits a strong preference for indole‐to‐tryptophan conversion. This process may influence serotonin levels, as tryptophan serves as a precursor for serotonin synthesis in enterochromaffin cells of the gut.^[^ ^17^ ^]^ These findings suggest an unexplored mechanism through which *Blastocystis* could modulate host tryptophan metabolism, potentially influencing gut microbial ecology and metabolic health. Future research should focus on elucidating the physiological consequences of *Blastocystis*‐mediated tryptophan metabolism, particularly in relation to metabolic disorders such as obesity, insulin resistance, and gut‐derived inflammation. Additionally, it is essential to investigate how different *Blastocystis* subtypes exert distinct effects on host metabolism, given that *BhTnaA* is highly conserved across various subtypes.

### 2.3 Immune Modulation via T‐Cell Differentiation

The gut microbiome is integral to the differentiation of immune cells such as T helper 17 (Th17) cells and Treg cells, both of which play pivotal roles in the pathogenesis and progression of hypertension.^[^ ^18^ ^]^ High dietary salt intake, a known risk factor for cardiovascular diseases, has been shown to deplete *Lactobacillus* populations and escalate the proportion of Th17 cells, subsequently increasing blood pressure in mice. Conversely, treatment with *Lactobacillus murinus* alleviates both experimental autoimmune encephalomyelitis and salt‐sensitive hypertension by modulating Th17 cell activity, likely through the production of indole‐3‐lactic acid, which has been shown to significantly reduce Th17 polarization in a dose‐dependent manner.^[^ ^19^ ^]^ On the other hand, Treg cells mitigate angiotensin II‐induced increases in blood pressure, vascular oxidative stress, inflammation, and endothelial dysfunction in experimental models by regulating the expression of the key transcription factor Foxp3 in the renal cortex.^[^ ^20^ ^]^

While many commensal bacteria contribute to immune homeostasis, *Blastocystis* subtypes appear to exert distinct, subtype‐specific effects on T cell differentiation, with potential implications for host metabolic health (Figure 1). Notably, *Blastocystis* ST7, a rare subtype with pathogenic potential, disrupts the balance between Treg and Th17 cells by favoring a pro‐inflammatory immune response.^[^ ^10^, ^21^ ^]^ Mechanistically, ST7 degrades tryptophan into indole‐3‐acetyldehyde (I3AA) via an aspartate aminotransferase‐dependent pathway. I3AA plays a dual role in modulating T cell function—enhancing T cell reactivity through TCR‐dependent mechanisms while preventing T cell exhaustion by downregulating PD‐1 expression. Furthermore, I3AA interferes with Treg function by suppressing TGF‐β signaling and CD103 expression, ultimately impairing immune tolerance. Additionally, I3AA antagonizes AhR signaling, a pathway essential for maintaining gut immune homeostasis, further exacerbating immune dysregulation.^[^ ^21^ ^]^ In contrast, *Blastocystis* ST4 colonization has been associated with an anti‐inflammatory immune profile, characterized by enhanced Treg differentiation and suppression of Th17 cells expressing IL‐17A in an experimentally induced colitis model. This effect appears to be mediated through ST4's capacity to regulate gut microbial composition and metabolic activity, reinforcing its potential role in maintaining immune balance.^[^ ^10b^ ^]^ These findings suggest that *Blastocystis* subtypes may exert subtype‐specific immune effects with significant implications for metabolic health. Future studies should aim to decipher the precise molecular pathways by which different *Blastocystis* subtypes influence immune cell differentiation and explore how these immune alterations impact host susceptibility to metabolic and inflammatory diseases.

### 2.4 Additional Metabolites Influencing Cardiometabolic Health

Beyond SCFAs and tryptophan metabolites, other microbiota‐derived metabolites, such as bile acids (BAs) and trimethylamine N‐oxide (TMAO), serve as crucial modulators of metabolic and cardiovascular health (Figure 1).^[^ ^22^ ^]^ BAs are synthesized and conjugated in the liver before being excreted into the large intestine, where gut microbiota convert primary BAs into secondary BAs through deconjugation and dehydroxylation.^[^ ^23^ ^]^ These secondary BAs interact with nuclear and membrane‐bound receptors such as farnesoid X receptor and Takeda G protein‐coupled receptor 5 (TGR5), both of which play critical roles in regulating lipid (cholesterol and triglyceride) and glucose balance, which are often disrupted in metabolic syndrome.^[^ ^24^ ^]^ Activation of TGR5 in enteroendocrine cells stimulates GLP‐1 production, enhancing insulin release from pancreatic β‐cells and improving glucose metabolism.^[^ ^23^ ^]^

TMAO, a well‐studied microbiota‐host co‐metabolite in cardiovascular disease contexts, is produced when gut bacteria metabolize dietary nutrients rich in choline, lecithin, and carnitine into trimethylamine, which liver enzymes then oxidize into TMAO.^[^ ^25^ ^]^ TMAO has been shown to induce hypertension and contribute to cardiac and renal fibrosis via the PRKR‐like endoplasmic reticulum kinase (PERK), reactive oxygen species (ROS), and calcium/calmodulin‐dependent protein kinase II (CaMKII) pathways.^[^ ^26^ ^]^ Although direct evidence linking *Blastocystis* to BAs and TMAO metabolism is currently lacking, its influence on the gut microbiota raises the possibility that it may modulate these metabolic pathways indirectly. Future studies should investigate whether *Blastocystis* can regulate BA and TMAO metabolism, elucidate the specific mechanisms underlying these interactions, and determine whether different *Blastocystis* subtypes exert distinct effects on these key metabolic regulators.

### 2.5 Integrative Role of *Blastocystis* in Host Metabolism

Although we have examined the effects of *Blastocystis* on SCFA production, tryptophan metabolism, and immune regulation as separate pathways, these processes are inherently interconnected, collectively shaping host metabolic health. The gut microbiota functions as a dynamic ecosystem, where microbial‐derived metabolites influence multiple signaling networks and immune responses in a coordinated manner (Figure 1). For instance, SCFAs have been implicated in modulating tryptophan metabolism through the AhR signaling pathway.^[^ ^27^ ^]^ AhR serves as a key sensor of microbial metabolites, orchestrating host responses to microbiota‐derived signals and infections.^[^ ^28^ ^]^ While SCFAs do not directly bind to AhR, they indirectly enhance AhR activation by inhibiting histone deacetylases (HDACs), thereby facilitating AhR transactivation.^[^ ^29^ ^]^ Notably, butyrate—a predominant SCFA—induces IL‐22 production in CD4⁺ T cells and innate lymphoid cells via AhR activation and upregulation of hypoxia‐inducible factor 1‐alpha.^[^ ^30^ ^]^ This intricate crosstalk suggests that *Blastocystis*‐induced shifts in SCFA production may have downstream effects on tryptophan metabolism and immune regulation through AhR modulation.

However, a fundamental question remains: does *Blastocystis* exert a direct regulatory effect on host metabolism, or does it function primarily as an ecosystem modulator by reshaping gut microbial communities? The observed subtype‐specific effects of *Blastocystis* on SCFA production, AhR activation, and immune cell differentiation suggest that its metabolic influence is more generally microbiome‐mediated. However, certain subtypes may also exert direct effects on host metabolism by producing unique metabolites that influence immune and metabolic pathways. This dual role of *Blastocystis*—as both an ecosystem modulator and a potential direct metabolic regulator—underscores the complexity of its interactions with the host. Future research should focus on unraveling the precise mechanisms by which *Blastocystis* interacts with host metabolism. Approaches such as metabolomics, targeted microbial engineering, and host‐microbiota interaction models will be critical in determining whether *Blastocystis* primarily exerts its effects through microbiome restructuring and functional outputs or if it possesses an intrinsic capacity to directly modulate host metabolic pathways.

### 2.6 Subtype‐Specific Effects of *Blastocystis* on Host Health

The impact of *Blastocystis* subtypes on host health is increasingly recognized, with accumulating evidence suggesting distinct functional roles. Some subtypes appear to confer beneficial metabolic and immune effects, while others have been linked to gut dysbiosis and inflammation. A study by Tito et al. using data from the flemish gut flora project identified a positive correlation between ST4 colonization and *Akkermansia*,^[^ ^31^ ^]^ a bacterial genus well‐documented for its metabolic benefits.^[^ ^32^ ^]^ *Akkermansia* has been shown to reduce serum triglycerides and alanine aminotransferase levels in high‐fat diet‐fed mice.^[^ ^33^ ^]^ Similarly, a proof‐of‐concept study found that administering live or heat‐inactivated *Akkermansia* to humans significantly reduced insulin and cholesterol levels in individuals with a high BMI compared to the untreated control group.^[^ ^34^ ^]^ Additionally, ST1 colonization was associated with increased *Akkermansia* abundance and conferred protection against DSS‐induced colitis in murine models.^[^ ^35^ ^]^ ST3 long‐term colonization also mitigated bacterial diversity loss following colitis induction, suggesting a potential role in gut inflammation recovery in a dinitrobenzene sulfonic acid‐induced colitis model.^[^ ^36^ ^]^

In contrast, ST7 has been implicated in gut dysbiosis and inflammatory pathologies. Studies have demonstrated that ST7 disrupts epithelial barrier integrity and induces the expression of pro‐inflammatory cytokines.^[^ ^37^ ^]^ Similarly, Yason et al. reported that ST7 not only suppresses the growth of commensal bacteria but also induces ulceration in the colon.^[^ ^38^ ^]^ Our epidemiological study in Singapore identified ST7 as the predominant subtype in diarrheal patients, with its presence correlating with decreased bacterial diversity and an increased abundance of *Enterobacteriaceae* and *Escherichia‐Shigella*.^[^ ^39^ ^]^ The mechanistic study further delineates the functional distinctions between ST4 and ST7.^[^ ^10b^ ^]^ ST4 colonization was found to enhance gut homeostasis by promoting beneficial bacteria, increasing SCFAs production, and inducing regulatory immune responses, as evidenced by a higher proportion of Foxp3⁺ and IL‐10⁺ CD4⁺ T cells. In contrast, ST7 exacerbated colitis severity by enriching pathogenic bacteria and triggering a pro‐inflammatory immune response characterized by increased IL‐17A⁺ and TNF‐α⁺ CD4⁺ T cells.^[^ ^10b^ ^]^ These findings highlight the subtype‐dependent effects of *Blastocystis* on microbial ecology, host metabolism, and immune function. While certain subtypes, such as ST4, may contribute to gut homeostasis and metabolic health, others, such as ST7, are associated with inflammatory pathologies.

## 3 Future Perspectives on *Blastocystis* Research

**Figure 2:**
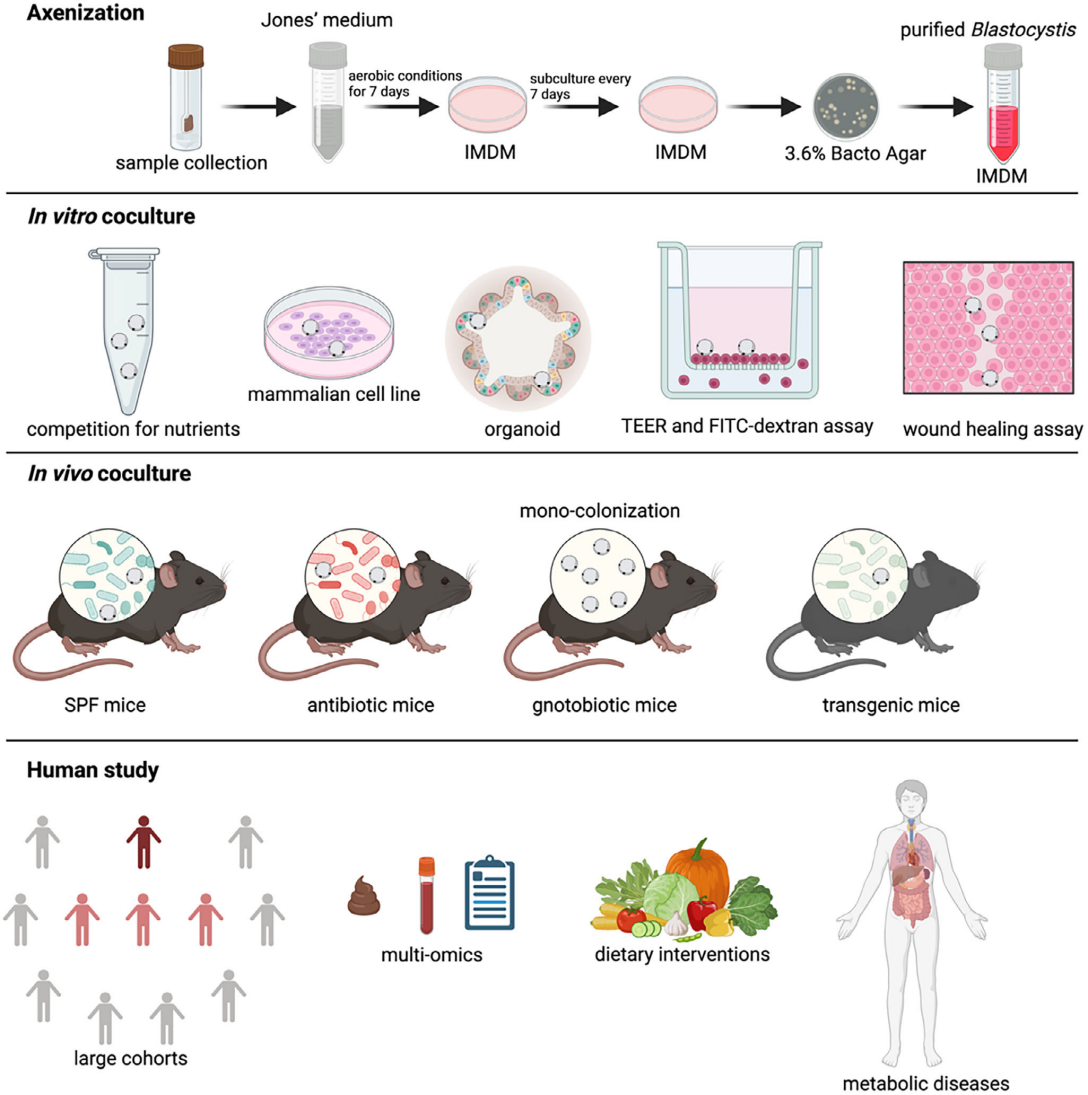
A systematic approach to investigate the potential influence of *Blastocystis* on cardiometabolic diseases. The framework includes the axenization of *Blastocystis*, followed by in vitro, in vivo, and clinical investigations to elucidate the mechanisms by which *Blastocystis* may impact host metabolism. Created with Biorender.com.

Although several potential mechanisms by which *Blastocystis* may influence cardiometabolic diseases have been proposed, direct causal relationships remain to be conclusively established. To advance our understanding of *Blastocystis's* impact on cardiometabolic health, we recommend a systematic research framework, building on over two decades of expertise in *Blastocystis* studies (**Figure** **2**).

### 3.1 *Blastocystis* Axenic Cultivation

The first step involves isolating *Blastocystis* strains through axenic cultivation.^[^ ^40^ ^]^ Positive stool samples, identified by microscopy or ​polymerase chain reaction, should initially be cultured under aerobic conditions in Jones’ medium for seven days. The cultures are then transferred to Iscove's Modified Dulbecco's Media (IMDM), supplemented with 10% horse serum and antibiotics (2000 µg mL^−1^ claforan, 500 µg mL^−1^ ampicillin, 100 µg ml^−1^ streptomycin, and 100 UI mL^−1^ penicillin) for further expansion. Subculturing every seven days is necessary until *Blastocystis* outcompetes bacterial populations. Colony morphology can be assessed by growing cultures on 3.6% Bacto Agar, with individual colonies subsequently expanded in IMDM. Identification of *Blastocystis* subtypes through whole‐genome sequencing or allele‐level resolution is essential for linking specific strains to pathogenic or beneficial traits, particularly for genetically diverse subtypes like ST7. While the culture conditions and growth rates are similar among different subtypes, they exhibit differences in morphology, surface coat structure (potentially facilitating bacterial nutrient acquisition), and genome size, all of which could influence their interactions with the gut microbiota.^[^ ^41^ ^]^

### 3.2 In Vitro Co‐Culture System

To explore interactions between *Blastocystis* and the gut microbiota under various nutritional conditions, we propose the use of in vitro co‐culture systems that include *Blastocystis* and bacterial co‐cultures under controlled conditions, as well as *Blastocystis* incubation with host cell lines, with or without bacterial co‐culture.^[^ ^42^ ^]^ These models allow for precise manipulation of environmental factors, enabling the study of *Blastocystis*’ effects on microbial growth, immune responses, and host‐microbiota interactions. Our previous studies have demonstrated differential subtype‐specific effects on microbial growth, with pathogenic ST7 inhibiting *Bifidobacterium longum* proliferation, whereas ST4 exhibited no such inhibitory effects in our in vitro co‐culture system.^[^ ^38^, ^42^ ^]^ Additionally, ST7 displays greater resistance to antimicrobial peptides and metronidazole compared to ST1 and ST4.^[^ ^43^ ^]^ Interestingly, ST4 was able to suppress lipopolysaccharide (LPS)‐mediated nuclear factor kappa B (NF‐κB) activation in THP1‐Blue cells, while ST7‐B enhanced LPS‐induced NF‐κB activation.^[^ ^44^ ^]^

Beyond bacterial interactions, mammalian cell lines and organoids are critical for mimicking the complex, multicellular environment of the gut, allowing for a more accurate assessment of how *Blastocystis* interacts with host tissues and other microbial communities. Assessments such as transepithelial electrical resistance and FITC‐dextran permeability assays will help evaluate the impact of *Blastocystis* on intestinal barrier functions. Additionally, wound healing assays will determine its effect on tissue repair processes. Further, co‐culturing *Blastocystis* with immune cell lines will help elucidate its role in shaping host immune responses, particularly its influence on pro‐inflammatory or regulatory cytokine production.

### 3.3 Pathophysiological Studies in Mouse Models

Establishing efficient and stable *Blastocystis* colonization in mouse models is crucial for investigating its pathophysiological effects.^[^ ^10^, ^35^ ^]^ While *Blastocystis* naturally colonizes humans and various animal species, its persistence in murine models remains challenging due to host‐specific microbiota dynamics. To overcome these limitations, optimized inoculation strategies are necessary, including multiple gavages, antibiotic pretreatment to reduce microbial competition, the use of germ‐free (gnotobiotic) mice for controlled microbial colonization, and co‐housing strategies to facilitate horizontal transmission. The choice of mouse model is critical, as microbiota composition directly influences *Blastocystis* colonization efficiency and its downstream effects on host metabolism and immune function.

Conventional mice provide a baseline for studying *Blastocystis* in a natural microbiota environment, while antibiotic‐treated mice facilitate colonization by eliminating competing bacteria, allowing for a more direct assessment of *Blastocystis*‐host interactions. Gnotobiotic models offer a controlled microbial landscape to investigate interactions between *Blastocystis* and specific bacterial consortia. Additionally, gene knockout models (such as AhR^−/−^, Foxp3^DTR^, GPR43^−/−^, and GPR41^−/−^) provide insights into how *Blastocystis* modulates immune responses, gut barrier function, and metabolic pathways, further elucidating its role in health and disease (**Table** **1**).

**Table 1:** Categorization of mouse models used in *Blastocystis* studies.

Mouse model	Microbiota complexity	Scientific use in *Blastocystis* studies	Refs.
Conventional mice	Normal, diverse microbiota, a list of known or potential pathogens is excluded (SPF)	Serves as a baseline for studying *Blastocystis* colonization in a natural gut environment and assessing its effects under normal physiological conditions.	[10, 35]
Antibiotic‐treated mice	Reduced microbiota due to antibiotic treatment	Used to deplete indigenous bacteria and facilitate FMT, enabling investigations into how *Blastocystis*‐altered microbiota affects host health. Additionally, facilitates *Blastocystis* colonization by depleting competing bacteria, allowing direct study of its effects on host physiology and microbiome interactions.	[10, 35]
Gnotobiotic mice	Germ‐free or colonized with a defined microbiota	Enables controlled studies on *Blastocystis* interactions with specific bacterial consortia, providing insights into its role in microbiome composition, metabolic pathways, and immune modulation.	[45]
Specific gene knockout mice	Microbiota composition varies depending on genetic modifications	Facilitates targeted investigations into host immune responses and metabolic pathways affected by *Blastocystis* by selectively knocking out genes involved in immunity, metabolism, and gut homeostasis.	[46]

### 3.4 Large Cohort Studies and Multi‐Omics Approaches

To establish robust connections between *Blastocystis*, dietary factors, and cardiometabolic health outcomes, future research should incorporate large‐scale, longitudinal cohort studies across diverse populations. While cross‐sectional studies have identified correlations between *Blastocystis* colonization and metabolic markers, the causality of these associations remains unclear. Detailed dietary records will be essential for determining how specific nutrients, such as fiber and fat, influence *Blastocystis* colonization and its metabolic effects. Longitudinal microbiome profiling using metagenomics and 16S rRNA sequencing will allow researchers to track *Blastocystis* dynamics over time and assess its impact on microbial community composition.

Integrating multi‐omics approaches—including metabolomics, proteomics, and transcriptomics—will provide deeper insights into how *Blastocystis* modulates host metabolism and immunity. Metabolomic analyses can clarify its role in SCFA production, tryptophan metabolism, bile acid conversion, and TMAO synthesis, linking these shifts to health outcomes. Immune profiling through cytokine assays, single‐cell RNA sequencing (scRNA‐seq), and flow cytometry will help determine subtype‐specific immunomodulatory effects. Advances in computational modeling and machine learning will further refine host‐microbe interaction networks, enabling the identification of protective versus pathogenic *Blastocystis* subtypes. These insights will be critical for developing targeted microbiome‐based interventions to improve metabolic and immune health.

This structured research framework is aimed at facilitating breakthroughs in our understanding of how *Blastocystis* interacts with the gut microbiome and subsequently influences cardiometabolic diseases. This approach could provide a causal linkage, potentially identifying novel therapeutic targets and interventions for managing these complex conditions and helping translate findings from experimental models to clinical trials, bridging the gap between research and clinical applications.

## 4 Concluding Remarks

While *Blastocystis* is strongly associated with healthy dietary patterns and improved cardiometabolic health, the causal nature and effect sizes of these associations remain unclear. Current evidence is largely correlational, necessitating controlled intervention studies to determine whether *Blastocystis* actively influences host metabolic regulation or simply reflects a healthier gut environment. Future research should focus on quantifying the magnitude of these associations, identifying potential confounders, and dissecting the specific microbial and host metabolic pathways through which *Blastocystis* may exert its effects. A combination of longitudinal cohort studies, dietary intervention trials, and mechanistic experiments will be crucial to elucidate the precise role of *Blastocystis* in host metabolism.

Increasing evidence from both in vitro and in vivo studies supports a commensal role for *Blastocystis* in the gut, suggesting its potential to modulate gut microbiota composition and orchestrate protective immune responses. The complex interplay between the gut and systemic systems, such as the gut‐brain and gut‐liver axes, underscores the potential for *Blastocystis* to influence overall host health. Nevertheless, critical questions remain regarding the feasibility of leveraging *Blastocystis* as a probiotic or the need for pharmaceutical interventions targeting its activity. Future research must determine whether *Blastocystis* colonization can be safely and reliably promoted in humans to harness its therapeutic potential. However, these findings should be interpreted with caution, as several studies indicate that certain pathogenic subtypes of *Blastocystis* may induce severe intestinal inflammation. As such, a balanced perspective is necessary when evaluating the role of *Blastocystis* in health and disease, emphasizing the need for further comprehensive studies to distinguish between its beneficial and potentially harmful effects.

## Conflict of Interest

The authors declare no conflict of interest.

## Author Contributions

L.D. wrote the original draft, and K.S.W.T. provided the review and editing. Figures were created with BioRender.com. All authors read and contributed to revisions of the work.
